# Characterization of the complete mitogenome of the estuarine benthic ribbon worm *Yininemertes pratensis* (Sun and Lu 1998) (Nemertea: Lineidae)

**DOI:** 10.1080/23802359.2021.1972858

**Published:** 2021-09-15

**Authors:** Sang-Hwa Lee, Yi Seul, Kwang-Soo Kim, Taeseo Park

**Affiliations:** a National Marine Biodiversity Institute of Korea, Seocheon, Republic of Korea; b Department of Biology Education, Konju National University, Kongju, Republic of Korea; c Division of Animal Resources, National Institute of Biological Resources, Incheon, Republic of Korea; d Overseas Biological Resources Team, National Institute of Biological Resources, Incheon, Republic of Korea

**Keywords:** *Yininemertes pratensis*, ribbon worm, complete mitogenome, Nemertea

## Abstract

The complete mitogenome of the estuarine ribbon worm *Yininemertes pratensis* (Sun and Lu 1998) (Nemertea: Lineidae) was sequenced for the first time in the present study. The total length of the newly sequenced mitogenome is 15,616 bp, and it includes 13 protein-coding, 2 rRNA, and 24 tRNA genes, in addition to a noncoding region of 571 bp. The phylogenetic position of *Y. pratensis* was examined through maximum-likelihood analysis using a concatenated dataset of 13 protein-coding genes from seven selected nemertean species. *Yininemertes pratensis* is placed within the pilidiophoran group and is closely related to *Lineus alborostratus* among the selected nemerteans. The newly determined mitogenome sequence will further our knowledge for future phylogenetic and ecological studies of this species.

Nemerteans, the so-called ribbon worms, are mostly marine benthic invertebrates that occur from the littoral zone to depths of 9000 m (Chernyshev and Maslakova 2011). They are carnivores, feeding on crustaceans, polychaetes, and mollusks, and nearly 1200–1300 nemertean species have been described worldwide (McDermott and Roe 1985; Kajihara et al. 2008; Chernyshev and Maslakova 2011; Kajihara 2017). Among these, only the estuarine ribbon worm *Yininemertes pratensis* (Sun and Lu 1998) (Nemertea: Lineidae) is known to be distributed in the estuaries of the Yangtze (Changjiang) River in China (Sun and Lu 1998) and the Han River in Korea (Park et al. 2019), which flows into the Yellow Sea. Explosive outbreaks of this species have occurred in recent years in the Han River estuary (Lee 2015; Noh 2019; Park et al. 2019). However, since the fundamental ecological characteristics, such as prey base, reproduction, and larval ecology, are uncharted, the exact reason for its massive proliferation remains unknown (Park et al. 2019).

In this study, *Y. pratensis* specimen was collected from the local fishers’ glass eel nets in the Han River estuary near the Haengju Bridge in Goyang-si, South Korea (37°36′08″N, 126°48′23″E). A voucher specimen has been deposited at the National Institute of Biological Resources (https://www.nibr.go.kr/, TP, polychaeta@gmail.com), Korea (NIBRIV0000409598).

Total genomic DNA was extracted using the DNeasy Blood & Tissue Kit (Qiagen, Hilden, Germany) according to the manufacturer’s protocol. Mitochondrial DNA was amplified using the REPLI-g Mitochondrial DNA Kit (Qiagen) to increase the concentration for the next-generation sequencing (NGS) analysis. The mtDNA library was prepared and sequenced on the Illumina Hi-Seq 2500 platform with 150 bp paired-end reads. A total of 6,264,724 raw sequences was trimmed using Trimmomatic program (Bolger et al. 2014). The cleaned reads were assembled in Geneious v. 9 and gene annotation were performed on web server, MITOS (Bernt et al. 2013). Furthermore, the phylogenetic position of *Y. pratensis* was determined in raxmlGUI 2.0 for macOS (Edler et al. 2021). A phylogenetic tree was reconstructed with the maximum-likelihood method using a concatenated dataset of 13 protein-coding genes from nine selected nemertean species.

The total length of the newly sequenced complete mitogenome of *Y. pratensis* is 15,616 bp (GenBank accession number: MW381012). The mitogenome contains 39 coding genes, including 13 protein-coding genes (PCGs), two ribosomal RNA genes, and 24 tRNA genes (with two more trnM genes), in addition to a non-coding region (NCR) of 571 bp. The gene order is similar to that of other heteronemertean mitogenomes data in NCBI, except added two trnM genes which were found between trnC and rrnS in *Y. pratensis* mitogenome.

The start codon of all the PCGs is ATG, except *nad1*, which starts with GTG. The stop codon of all PCGs is TAA (*cox2*, *cytb*, *nad4*, *nad4L*, and *nad6*) or TAG (*cox1*, *cox3*, *atp6*, *atp8*, *nad2*, *nad3*, and *nad5*), except *nad1*, which terminates in T.

The result of phylogenetic analysis shows that *Y. pratensis* is placed within the pilidiophoran species, and is closely related to *Lineus alborostratus* Takakura, 1898 among compared nemerteans (Figure 1). The newly sequenced *Y. pratensis* mitogenome will be useful not only for further taxonomic and phylogenetic studies but also for ecological studies aimed at understanding the reason for the massive proliferation of this species in the Han River estuary.

**Figure 1.**
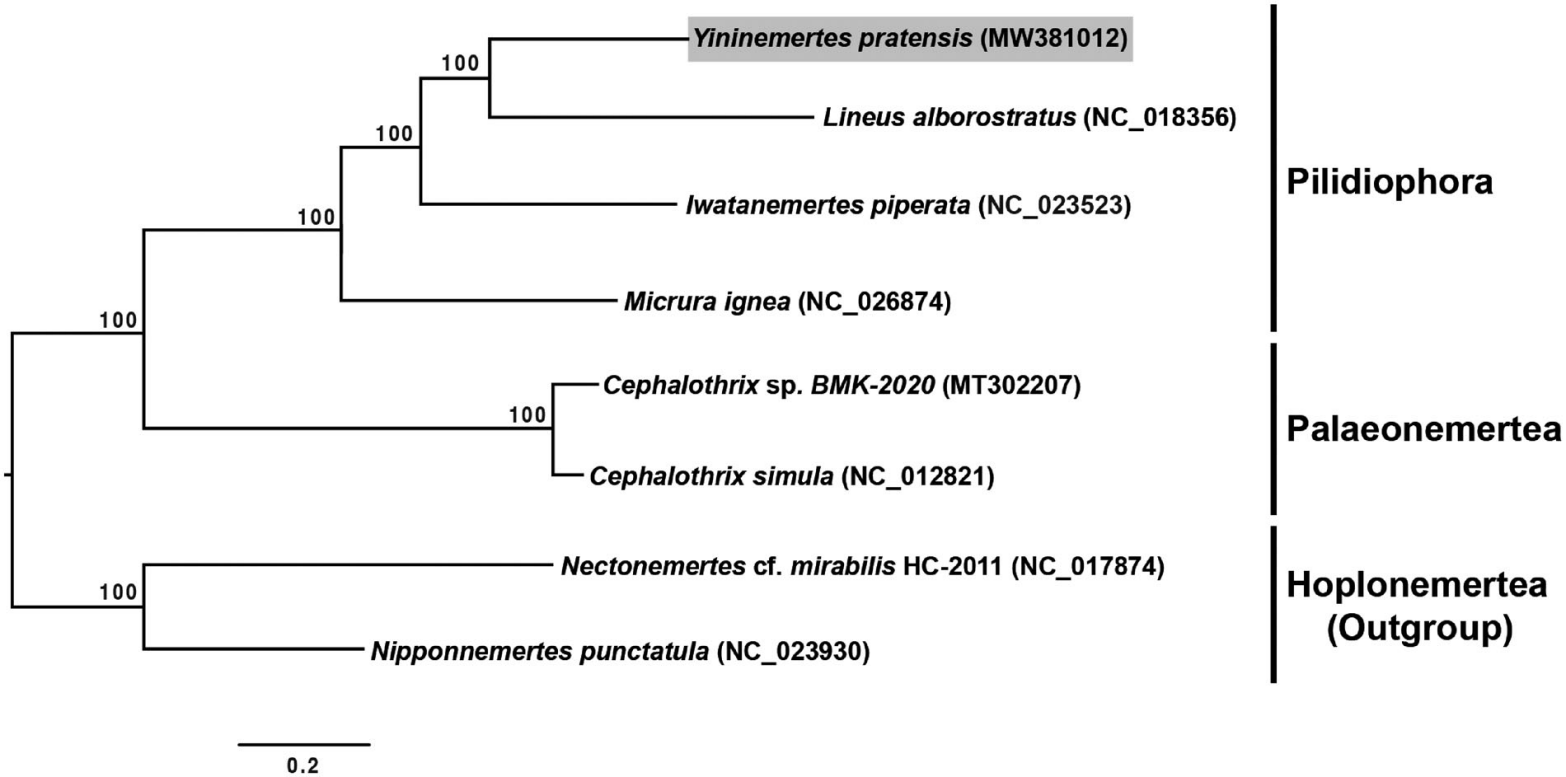
Maximum-likelihood (ML) tree constructed using the concatenated dataset of 13 protein-coding genes based on eight mitogenome sequences, including the *Yininemertes pratensis* mitogenome sequence from the present study. In total, 10,000 bootstrap replicates were performed. Letters in parentheses indicate GenBank accession numbers.

## Data Availability

Mitogenome data supporting this study are openly available in GenBank at nucleotide database, https://www.ncbi.nlm.nih.gov/nuccore/MW381012.1, Associated BioProject, https://www.ncbi.nlm.nih.gov/bioproject/?term=PRJNA740112, BioSample accession number at https://www.ncbi.nlm.nih.gov/biosample/?term=SAMN19820787 and Sequence Read Archive at https://www.ncbi.nlm.nih.gov/sra/?term=SRR14887747.
